# Identification and Antimicrobial Susceptibility Testing of Anaerobic Bacteria: Rubik’s Cube of Clinical Microbiology?

**DOI:** 10.3390/antibiotics6040025

**Published:** 2017-11-07

**Authors:** Márió Gajdács, Gabriella Spengler, Edit Urbán

**Affiliations:** 1 Department of Medical Microbiology and Immunobiology, Faculty of Medicine, University of Szeged, 6720 Szeged, Hungary; spengler.gabriella@med.u-szeged.hu; 2 Institute of Clinical Microbiology, Faculty of Medicine, University of Szeged, 6725 Szeged, Hungary; urban.edit@med.u-szeged.hu

**Keywords:** anaerobic bacteria, susceptibility testing, methodology, antimicrobial resistance, MALDI-TOF MS, *Bacteroides fragilis* group, *Clostridium* spp. taxonomy, metronidazole, β-lactams

## Abstract

Anaerobic bacteria have pivotal roles in the microbiota of humans and they are significant infectious agents involved in many pathological processes, both in immunocompetent and immunocompromised individuals. Their isolation, cultivation and correct identification differs significantly from the workup of aerobic species, although the use of new technologies (e.g., matrix-assisted laser desorption/ionization time-of-flight mass spectrometry, whole genome sequencing) changed anaerobic diagnostics dramatically. In the past, antimicrobial susceptibility of these microorganisms showed predictable patterns and empirical therapy could be safely administered but recently a steady and clear increase in the resistance for several important drugs (β-lactams, clindamycin) has been observed worldwide. For this reason, antimicrobial susceptibility testing of anaerobic isolates for surveillance purposes or otherwise is of paramount importance but the availability of these testing methods is usually limited. In this present review, our aim was to give an overview of the methods currently available for the identification (using phenotypic characteristics, biochemical testing, gas-liquid chromatography, MALDI-TOF MS and WGS) and antimicrobial susceptibility testing (agar dilution, broth microdilution, disk diffusion, gradient tests, automated systems, phenotypic and molecular resistance detection techniques) of anaerobes, when should these methods be used and what are the recent developments in resistance patterns of anaerobic bacteria.

## 1. Introduction

Anaerobic bacteria have been implicated in a wide range of infectious processes. As an integral part of the human microbiome, these microorganisms can be found in different anatomical sites and they can be responsible for a plethora of infections that may be serious or life-threatening [1,2,3,4]. For this reason, anaerobes are often categorized by the body site(s) where they occur, the infections they are associated with, although they are most frequently classified by their microscopic morphology (rods or cocci) and their Gram-staining (for a summary of the most important genera of anaerobic bacteria, see Table 1) [3,5,6]. Infections caused by anaerobes can also be divided according to the origin of the microorganisms. Exogenous anaerobic infections are predominantly caused by Gram-positive spore-forming bacilli (*Clostridium* spp.; *C. difficile* being an exception to this rule), these microorganisms are the causative agents of serious infections like visceral gas gangrene, lockjaw, myonecrosis and botulism. These infections are monobacterial in almost all cases and the effects of bacterial toxins are the prime causes of these pathologies [1,7]. In these cases—although microbiological evaluation is still recommended—diagnosis is mostly reached based on the characteristic clinical symptoms—on the other hand, the overwhelming majority of anaerobic infections are of polymicrobial nature, due to multiple causative agents, involving both obligate aerobes, facultative and obligate anaerobes, in addition, the vast majority of these organisms originate from the normal flora of the skin and mucous membranes [1,2]. These infections commonly involving anaerobes including but not limited to: actinomycosis, root canal and other odontogenic infections, chronic otitis media, chronic sinusitis, aspiration pneumonia, peritonitis and appendicitis, endometritis, salpingitis, necrotising fasciitis, osteomyelitis, septic arthritis, anaerobic cellulitis, infections of wounds and ulcers, abscesses of the head and neck region, lungs, intra-abdominal organs, liver, fallopian tube, ovary and adjacent pelvic organs etc. The most frequent causative agents of these infections are Gram-negative (e.g., *Bacteroides fragilis* group, *Porphyromonas* spp., *Prevotella* spp. and *Fusobacterium* spp.) and Gram-positive (non-spore forming) bacilli (e.g., *Actinomyces* spp., *Propionibacterium* spp., *Eubacterium* spp., *Bifidobacterium* spp.) as well as Gram-positive cocci (e.g., *Anaerococcus* spp., *Atopobium* spp., *Finegoldia* spp., *Peptostretococcus* spp., *Sarcina* spp.) [2,4,8]. The involvement of anaerobic infections varies based on the anatomical site but their significance should not be underrated, as they are present in around 5–10% of bacteraemia, 89% of brain abscesses, 93% of post-surgery abdominal infections, 76% of thoracic empyemas, 95% of diabetic foot infections, 52% in chronic sinusitis, 30–40% of all bite wound infections and 63% in septic abortions [2,4,6,9,10,11,12,13]. Their importance was further underlined when their roles in the pathomechanism of bacterial vaginosis (BV) and pelvic inflammatory disease (PID) were explained [14,15], also with the emergence of antibiotic-associated diarrhoea (AAD) and toxic megacolon caused by toxin producing *Clostridium difficile* (especially the hyper virulent ribotype 027), a significant nosocomial pathogen which is very hard to eradicate, both from a hospital hygiene perspective and therapeutic setting [16,17,18,19]. The clinical significance of anaerobes and the interest concerning these bacteria is steadily increasing, with new species described in various infectious processes almost every day, especially in immunocompromised patients [20]. 

A range of factors are known to increase the likelihood of anaerobic (mixed) infections, most of which are related to either processes that damage the mucous membranes or conditions reducing the oxygen levels of tissues. Some of these predisposing factors include diabetes, angiopathies of different origin, malignancies, animal and human bites, wounds contaminated with soil, burns, trauma, surgical interventions (both minor and major), foreign bodies, immunosuppression due to AIDS or drug therapy (corticosteroids, cytotoxic agents), procedures involving fine needle aspiration among others [2,4]. In some cases, other infections can be predisposing factors as well (e.g., the correlation between the causative agent of infectious mononucleosis (Epstein-Barr virus; EBV) and Lemiere’s syndrome (necrobacillosis caused by *Fusobacterium necrophorum*) [21,22]. Aminoglycoside monotherapy is also associated with the selection and overgrowth of anaerobes (since these microorganisms are intrinsically resistant), which may lead to secondary infections in a healthy or immunocompromised host [23].

In the recent years, considerable changes occurred in the taxonomy of anaerobic bacteria thanks to the developments in molecular methods (DNA hybridization, G + C nucleotide content analysis) and sequencing technologies (next generation sequencing). Anaerobic Gram-negative bacilli (AGNB) were mostly affected, although other genera experienced some changes as well, e.g., in the case of the genus *Peptostreptococcus* (several species were reclassified to other genera, e.g., *Anaerococcus, Finegoldia, Parvimonas, Peptoniphylus*) or the proposal to change *Clostridium difficile* to *Clostridioides difficile* to name a few under the umbrella of Gram-positive anaerobes [24,25,26,27,28,29,30,31]. There is debate however, on the role of these changes in the healthcare setting and whether clinicians should familiarise themselves with the new nomenclature [3,25]. 

Anaerobic bacteria are significant constituents of the normal microbiome of humans, so much so that they outnumber aerobic microorganisms 10:1–1000:1 in the normal flora of the skin and gut, additionally, they are important in keeping the physiological homeostasis of the oral cavity and the genito-urinary tract of females [3]. These bacteria are passed from mother to the new-born during vaginal birth, becoming an important part of our microbiota in the very early stages of life [32,33].

The colon accommodates the largest population of bacteria in the human body (10^10^–10^12^/g), with most of these organisms being anaerobes [33]. Some hypothesize that we should consider the microbiota of our gastro-intestinal tract as an “organ” by itself, critical in maintaining health and preventing various diseases [34]. There are also reports drawing parallels between the state of the gut microbiome and obesity [35], type I diabetes [36], several types of cancer [37,38,39,40], psychiatric disorders and depression [41,42,43], attention deficit hyperactivity disorder [44] and even autism [45]. It has been described that commensal anaerobes have a critical role in regulating the immune functions of the large intestine, protecting us against their pathogenic counterparts, contributing to colonisation resistance (e.g., the metabolising of bile salts to prevent the overgrowth and spore formation of *C. difficile*) [34]. From another point of view, the composition of the microbiota is also significant due to inter-species and intra-species horizontal gene transfer (HGT) of various resistance determinants [46,47].

## 2. Cultivation and Identification of Anaerobes

Anaerobic bacteria (according to a definition by Syndey M. Finegold) are microorganisms that are unable to grow on solid media in an atmosphere containing 18% O_2_ and 10% CO_2_ [2]. They vary in their level of tolerance towards atmospheric oxygen and toxic oxygen species (i.e., hydrogen peroxide [H_2_O_2_], superoxide anion [O_2_^−^], hydroxyl radical [OH*] and singlet oxygen [O_2_*]), based on the presence or absence of the enzymes required to eliminate them (superoxide dismutase; SOD, catalase and peroxidase) [2,5,48,49]. Obligate anaerobes (e.g., *C. perfringens*) do not tolerate the effects of oxygen exposure (damage through oxidation of lipids, inactivation of enzymes and direct effects on the genetic material of these microorganisms). On the other hand, aerotolerant anaerobes (e.g., *Cutibacterium/formerly known as Propionibacterium/acnes*) possess superoxide dismutase and peroxidase, therefore oxygen does not have a detrimental effect on them (note: strain-to-strain differences in aerotolerance is common even among strict anaerobes) [48,50]. The tolerance of these bacteria to oxygen is further influenced by certain environmental conditions (*C. perfringens* can withstand higher oxygen levels at lower pH, e.g., in an abscess). Microaerophilic bacteria (e.g., *Helicobacter pylori, Campylobacter jejuni*) and anaerobes should be discussed separately. Although their growth requires lower levels of oxygen than atmospheric levels (in addition to this, some of these microbes are capnophilic, which means they thrive in higher levels of CO_2_) but they ultimately need it for survival, since they do not have the ability to ferment, in contrast to anaerobic bacteria using fermentation as a primary source of ATP and cellular energy [51,52].

When it comes to the cultivation of anaerobes in a clinical microbiology laboratory, accepting only the samples appropriate for such purposes is of utmost importance. Specimen collection should be done using suitable equipment, while making sure that the sample is not contaminated with the resident flora of the body site in question. If sample processing is taking place somewhere else (e.g., is sent to a reference laboratory) adequate precautions need to be met for the use of anaerobic transport media and storage. The following specimen types should be considered for cultivation: samples taken from normally sterile body sites (e.g., blood cultures, cerebrospinal fluid samples, synovial fluid, samples from the chest and abdominal cavity), surgical discharge (or sample taken from the surgical site), samples from deep wounds (e.g., animal bites), abscesses of different anatomical localisation (liver, brain, lungs), perioral and gingival samples, samples from diabetic foot ulcers, respiratory secretions taken with a double-lumen device, urine samples (taken only with suprapubic aspiration) and faecal samples (in the suspected case of *C. difficile* infection). The best specimens for anaerobic cultivation are those taken using a needle and a syringe. Any other sample type received should not be accepted for anaerobic processing since they do not meet the requirements (risk of oxygen exposure: false negative result; contaminants: false positive results and/or cultivation of microorganisms with no clinical significance) [5,6,49,53,54,55].

Adequate laboratory conditions are necessary for the cultivation and diagnosis of anaerobic bacteria, the most important of which is a suitable method to anaerobiosis (stable anaerobic environment for cultivation) and the availability of pre-reduced anaerobically sterilised (PRAS) media. Some *physical* (McIntosh-Filde’s jar), *chemical* (e.g., basic pyrogallol to bind atmospheric oxygen) and *biological* (Fortner’s method; co-cultivation with a strong aerobic metabolism in a split Petri-dish, e.g., *Serratia marcescens*) methods are now only of historical significance. Depending on the size of the clinical laboratory and the number of incoming isolates, GasPak sachets, anoxomat systems or anaerobic chambers (glove boxes) are used, although the availability of some of these is limited to anaerobic reference laboratories [5,6,49,53,56].

Anaerobic bacteria are fastidious microorganisms, culturing them requires nutrition-rich media and some of these include components that allow for the selective growth of these bacteria. Examples include liquid enrichment media like Holman-broth (chopped meat and carbohydrates) and thioglycolate-broth and a variety of pre-reduced anaerobically sterilised solid media (anaerobic blood agar, Brucella blood agar supplemented with 5% lysed horse blood, 5 μg/mL hemin and 1 μg/mL Vitamin K1 [BBA], Schaedler blood agar [SCS], Bacteroides-bile-esculin agar [BBE], kanamycin-vancomycin leaked blood agar [KVLB or LKV], phenylethyl alcohol agar [PEA], cycloserine-cefoxitine fructose agar [CCFA], colistin-nalidixic acid media [CNA], egg-yolk agar [EYA]) [5,49,57].

A variety of methods can be used for the identification of anaerobic isolates, from presumptive identification (based on growth characteristics, colony morphology, susceptibility to given antibiotics, presence or absence of fluorescence, classical biochemical tests and spot-tests) to commercial kits (e.g., Vitek 2 ANI card, API 20A, RapidID 32A: bioMérieux, Fr., MicroScan, BBLCrystal Anaerobe ID: Beckton Dickinson, UK) [49,58]. When it comes to the biochemical ID, it is important to be aware of the enzymes we target in an assay. Reactions involving *constitutive* enzymes can be included in rapid identification kits, while in the case of *inducible* enzymes, the presence of the substrate and additional time for the reaction is required. An additional method, specific for these bacteria, is available: using gas-liquid chromatography (GLC), a rapid, accurate and reliable identification method of anaerobes is possible, based on the alcohols, organic acids and short-chain fatty acids they produce [59]. This method can also be used to differentiate between aerobic and anaerobic infections (e.g., from a haemoculture), many laboratories however, do not possess the instruments required to carry out this assay [60,61]. For further reading on the phenotypic methods concerning anaerobic bacteria, see the *Wadsworth-KTL Anaerobic Bacteriology Manual*, which is considered as a reference in the phenotypic identification of these microorganisms [5].

The introduction of molecular methods, array-based systems and novel technological advancements in clinical microbiology, such as whole-genome sequencing (WGS) and matrix-assisted laser desorption/ionization time-of-flight (MALDI-TOF) mass spectrometry (which utilizes the spectra of conserved ribosomal proteins for identification) has revolutionised the detection and correct identification of anaerobic bacteria. Although the development of databases containing bacterial spectra, required for identification is still underway (with projects like the ‘European Network for Rapid Identification of Anaerobes’ [ENRIA] leading the charge), MALDI-TOF MS changed the face of diagnostic bacteriology in the last decade, both in the case of aerobes and anaerobes [62,63,64,65,66,67,68,69,70,71]. Whole-genome sequencing is a novel technology used for culture-independent diagnostic testing (CIDT). WGS is considered the “golden standard” of bacterial species identification, based on the complete sequencing of their genetic material and then, comparing the data to reference genomic libraries based on bioinformational methods. Nevertheless, while there are some commercial MALDI-TOF MS systems available, specifically for all purposes (including anaerobes and fungi) of routine clinical microbiology (e.g., VITEK MS: bioMérieux, Fr., MALDI Biotyper or Microflex LT of Bruker-Daltonics, Gr.) [72,73], sequencing technologies are-in most cases-still in their developmental/experimental phase (and not necessarily well characterized for anaerobes) [29,62,74,75,76]. Besides the growing interest in the research related to anaerobic bacteria, the description and verification of new species has also been facilitated by the introduction of these new technologies-although such research is limited to reference laboratories with the appropriate facilities [20,77,78].

Compared to the processing time of aerobic bacteria or facultative anaerobes the workup of anaerobic isolates usually takes much longer. For this reason, continuous communication between the clinicians and the diagnostic lab is crucial. The laboratory should supply any clinically relevant information in a precise and timely manner, while the feedback of the physicians is also important, since based on the information about the symptoms and the clinical picture, the lab can narrow down (or include more specific tests) the list of the possible causative agents. If anaerobic bacteria were present at the infection site and we failed to detect them, it can often lead to inappropriate therapeutic choices and clinical failure [8].

## 3. Examples of Clinically Relevant Anaerobic Bacteria

### 3.1. Members of the Bacteroides Fragilis Group

Anaerobic Gram-negatives include some of the most important human pathogens among the group of anaerobic bacteria. The group is included in the phylum *Bacteroidetes* (together with pathogens like *Capnocytophaga canimorsus*, *Porphyromonas gingivalis*, *Prevotella melaninogenica* and *Tannerella forsythia*) [6,50]. Together with the taxonomy of other anaerobic species, the classification of *Bacteroidetes* has undergone major changes due to findings of studies related to molecular, sequencing and other related metagenomic studies. The most profound changes can be seen in the genus *Bacteroides*, in which mostly Gram-negative, bile-resistant bacilli remained (Table 2). Species that were saccharolytic, bile-sensitive (pigmented [formerly known as *B. melaninogenicus*] or nonpigmented) were relocated to the genus *Prevotella*, while the asaccharolytic, pigmented species to the genus *Porphyromonas* [24]. The previous classification of the group was as an order of subspecies (e.g., *B. thetaiotaomicron* was previously known as *B. fragilis* ssp. *thetaiotaomicron*) but later was reclassified based on studies related to the DNA homology of the respective species. The members of the *Bacteroides* spp. are important constituents of the faecal bacteria flora of adult humans, they have an active role in the metabolism of bile salts, nitrogen-containing substrates and various carbohydrates and polysaccharides (e.g., arabinogalactan, starch, pectin, xylan) [6,23,50].

The antibiotic resistance levels among anaerobes is the best characterized in *Bacteroides* isolates and according to the relevant literature, the species of the genus have the highest levels of antibiotic resistance and the most numerous number of antibiotic resistance mechanisms among all human pathogenic anaerobic bacteria (described in detail in Section 5) [79,80]. It is important to note that resistance levels of these bacteria are in inverse relationship with their incidence and clinical significance: *P. distasonis* and *B. thetaiotaomicron* (13–23% of *Bacteroides* isolates) exhibit the highest levels of resistance, while the strains of *B. fragilis* is generally the most susceptible species in the *B. fragilis* group.

*B. fragilis* is a pleomorphic (with varying sizes of 1.5–6 µm), non-pigmented, non-motile, encapsulated, Gram-negative rod. The species is catalase-positive (which is not common for anaerobe bacteria), indole-negative and the clinical isolates are growing well in the presence of 20% bile (bile resistant). They do not produce fluorescence under UV-light and they are resistant to vancomycin, kanamycin and colistin antibiotic disks, which can be used for their presumptive identification. *B. fragilis* is the most frequently isolated member of this group and although they only represent 2–5% of the species isolated from the fecal flora, it accounts for >50% of the clinical isolates of the *Bacteroides* genus and is responsible for around 80% of infections caused by the genus [50].

Certain strains termed enterotoxigenic *B. fragilis* (ETBF) produce a metalloprotease enterotoxin, which is coded by the *bft* genes on the *B. fragilis* pathogenicity island (BfPAI). The disease was first described in new-born lambs and calves, nowadays it is acknowledged as a significant cause of a self-limiting diarrheal disease in adults. Certain reports drew the conclusions that colonisation with enterotoxigenic *B. fragilis* is also associated with higher risk for colorectal cancer and irritable bowel syndrome (IBS), however there is still debate over the conclusions of these results. The proper diagnosis of enterotoxigenic *B. fragilis* infection is laborious, detection of the toxin genes (*bft*1–3) by polymerase chain reaction or the determination of the biological activity of the BFT protein in a cell culture assay (for this purpose, only those cell lines can be used, which have the ability to polarize in vitro, e.g., HT-29, Caco-2, HCT-8, MDCK, etc.) is needed. Animal models have also been described to verify the presence of the enterotoxin of *B. fragilis*, however the use of these models is only appropriate in a research setting [50,81,82]. A very important virulence factor of these bacteria is the presence of the capsule (consisting of two, well characterised polysaccharides), the significance of which in abscess formation was proven earlier in animal studies [83]. Additional virulence determinants of this species include the presence of various fimbriae and adhesions, which help the bacteria to adhere to matrix proteins of the host [50]. The most significant role of *B. fragilis* as an infectious agent is thought to be in anaerobic bacteraemia, where the associated mortality is estimated to be around 19% (60% if left untreated) [84,85].

*B. fragilis* is frequently termed as the “anaerobic *Escherichia coli*” due to the many parallels in the characteristics of these microbes: (i) both are bile-resistant Gram-negative rods; (ii) both colonise the colon and have integral roles as a part of the microbiota; (iii) both have the proclivity to become multidrug resistant pathogens through a variety of resistance mechanisms (iv) some strains produce enterotoxin (enterotoxigenic *B. fragilis* vs. enterotoxigenic *E. coli*) and capsule (in the case of extra-intestinal pathogenic *E. coli* strains). The presence of this species was associated with pathogen synergy, it has been described that the presence of *B. fragilis* and *E. coli* in mixed intra-abdominal infections enhanced the anti-complement environment of the infection site through bacterial virulence factors [86].

### 3.2. Members of the Clostridium Genus

Anaerobic bacteria were traditionally classified into the *Clostridium* spp. based on the following characteristics: (i) their staining result was Gram-positive (ii) being strict anaerobic (iii) formation of characteristic endospores (their position is useful in species identification; *C. tetani* has round, terminal spores, *C. botulinum* and *C. difficile* forms oval, subterminal spores, *C. perfringens* has central spores with oval shape, i.e., in the appropriate environment) (iv) inability to reduce sulphates (SO_4_^2−^) into sulphites (SO_3_^2−^) [6,7]. Nevertheless, the classification of these bacteria based on these phenotypic criteria has become difficult, since some species do not form spores or only do in specific conditions (e.g., *C. perfringens*, *C. ramosum*), some are uncharacteristically tolerant to atmospheric oxygen (e.g., *C. tertium*, *C. histolyticum*), while others consistently present as Gram-negative after staining (*C. ramosum*, *C. clostridiforme*). Due to these discrepancies, it’s not surprising that the reclassification of multiple species to various other genera (e.g., *Butyrivibrio*, *Dendrosporobacter*, *Enterocloster*, *Eubacterium*, *Faecalicatena*, *Lacriformis*, *Sedimentibacter*) has been proposed, based on 16S rRNA gene sequencing data [87,88]. 

Species of the *Clostridium* spp. are transient or permanent members of the normal flora of the gastro-intestinal tract and skin of animals and humans alike. Their spores naturally occur in the soil, their presence is facilitated with the use of manure as fertilizer. It is important to point out that many clinical specimens could contain *Clostridium* spp. as accidental contaminants, not involved in the disease process. It is up to the clinician to decide, whether the presence of these bacteria has any clinical significance, based on the symptoms of the patient, the presence of other microorganisms of pathogenic potential, frequency of isolation of the species and local epidemiology. The adequate management of clostridial infections involves administering antibiotic therapy (e.g., penicillin, vancomycin, metronidazole), with tissue debridement, where it is necessary. In some cases (like tetanus) toxoid immunization and antitoxin therapy are also important tools for clinicians [1,2,6,7].

The pre-requisite for clostridial wound infections is trauma to host tissue, to lower the oxidation-reduction potential and provide an environment suitable for anaerobic growth, followed by accidental contamination of the wound. Clostridial wound infections usually are polymicrobial in nature, because the sources of wound contamination (faeces, soil) is polymicrobial, involving a variety of other bacterial isolates (e.g., *Bacillus* spp., *Bacteroides* spp., *Escherichia* spp., *Proteus* spp., *Staphylococcus* spp.) [6,7]. All clostridial species involved in human diseases (see Table 3.) produce some kind of protein exotoxin (e.g., tetanus toxin, botulinum toxin) which plays an important role in the corresponding pathologies. Based on the pathogenesis of the disease caused by these microorganisms, they can be divided into the group of *histotoxic clostridia* (or gas-gangrene clostridia: *C. perfringens*, *C. novyi*, *C. septicum*, *C. histolyticum*) and neurotoxic clostridia (lockjaw/tetanus: *C. tetani*; botulism: *C. botulinum*, *C. baratii*, *C. butyricum*). Toxin-producing *C. difficile* strains represent an additional group, as the causative agents of antibiotic-associated diarrhoea (AAD), pseudomembranous colitis and toxic megacolon [6,7]. 

Various strains of *C. perfringens* produce potent necrotizing and haemolytic toxins and enzymes, based on this quality, these strains can be classified into types A-E. All types produce the α-toxin (the principal virulence factor of this species), which is a lecithinase that destroys the membranes of red and white blood cells and of surrounding tissue cells. The production of α-toxin can be detected by the Nagler-reaction, using egg-yolk agar (EYA). Other toxins of *C. perfringens* have a haemolytic, necrotizing and cardiotoxic effect. Among its other enzymes, the most important are collagenase, hyaluronidase and deoxyribonuclease. Some of the type A strains produce a heat-labile enterotoxin during sporulation (often occurring in the small intestine, at a relatively high pH), binds to the membrane of the epithelial cells of the small intestine, causing diarrhoea. The activity of this toxin is greatly increased by digestion with trypsin [6,7,89]. Tetanospasmine (or tetanus toxin, which is a typical AB-type exotoxin), the most important virulence factor of *C. tetani*, is released by dissolution the microbial cell. The toxin can reach the spinal cord, partially through the blood stream and the lymphatic system but mainly and typically by retrograde axonal transport through the peripheral neurons. The active A subunit, which is a zinc-endopeptidase, inhibits the release of inhibitory neurotransmitters (glycine and γ-aminobutyric acid) from inhibitory neurons. Thus, the proliferation of acetylcholine generates a continuous activation resulting in spastic, convulsive paralysis (causing the characteristic symptoms of the disease) [6,7].

The botulinum toxin (or botulotoxin) is the exotoxin of *C. botulinum*. It is produced as a consequence of lysogenic conversion, seven different (A-G) structurally different variants are known, of which the A, B, E and F-type toxins have been reported in human diseases. For any symptom to occur, whether or not it is caused by preformed toxins (classical food poisoning) or the effect of toxins produced by the microorganism in the body (infant and wound-botulism), the botulinum toxins is responsible. Botulinum toxin is a very powerful poison and in contrast to other toxins, protein-digesting enzymes of the intestinal tract do not hydrolyse it but it is heat-labile, meaning that the proper treatment of the food (at 80 °C for at least 10 min) inactivates it. In many respects, it is similar to the tetanus toxin (it is also an AB-type zinc-endopeptidase). However, unlike tetanus toxin, the botulinum toxin does not migrate retrograde to the neuronal cell, after entering the neuromuscular junctions through endocytosis. The toxin cleaves the protein required for acetylcholine release in the presynaptic neuron, the release of acetylcholine is thus omitted and in the absence of the neurotransmitter, the distinct symptoms of flaccid paralysis can be observed [6,7,90].

Similarly to other members of the genus, exotoxins are responsible for the symptoms of *C. difficile* infection as well. The A-toxin is an enterotoxin, which causes injury to intestinal cell-cell connections with subsequent increase in the permeability of the intestinal epithelium and fluid secretion. It also enhances the migration white blood cells, inducing a local inflammatory reaction. B-toxin is a cytotoxin that damages the cytoskeleton of the cells by disorganizing intracellular actin filaments. The two toxins have a synergistic effect but the A-toxin negative strains seem to be just as capable of causing illness and even causing hospital epidemics. The third toxin is the so-called binary toxin (or CDT), the exact mode of action of which is unknown, although it has been recognized that strains producing binary toxin are associated with higher mortality and produce A and B toxins in much larger quantities than CDI-negative strains [6,17,18].

## 4. Antimicrobial Susceptibility Testing of Anaerobes

### 4.1. General Considerations

According to the international guidelines, the susceptibility testing of anaerobic bacteria is very expensive, time-consuming and requires experienced laboratory staff, testing every patient’s isolate received in a routine laboratory is not warranted. There are some diagnostic institutions (e.g., in low-income countries, with no technical capabilities) where even the cultivation of these microorganisms is a challenge. In these cases, isolates are usually sent to higher-tier facilities or a national anaerobic reference laboratory. Based on current recommendations, susceptibility testing should be performed in the following cases: (i) infections of a serious and life-threatening nature (e.g., in endocarditis, bacteraemia, abscesses involving the brain) [2,10,84]; (ii) relapsing infections or infections that did not respond to empirical therapy at all; (iii) when the antibiotic therapy needs to take place for an extended time period (e.g., infections involving bones, joints, implanted devices or grafts) [12,13]; (iv) there is limited or no available data on the susceptibilities of the given organism; (v) there is a known pattern of resistance against the antimicrobial agent by the microorganism [79]; (vi) the causative agent is a particularly virulent anaerobe (see Table 4) with unpredictable resistance [8,23]; (vii) the microorganism was isolated in pure culture and/or from a normally sterile body site (see Section 2) [8,23]. Besides the abovementioned points, susceptibility testing should be performed wherever it is possible, for epidemiological purposes and to guide the choice of the therapeutic agents [8,91,92]. This aspect of surveillance (both locally and globally) needs to be reiterated, since recommendations on first-line agents of therapy is usually based on similar data (Table 5). For example, due to the high level of resistance, cefoxitin and cefotetan are not advised as first-line drugs and clindamycin has been completely removed from such recommendations (although it can still be used in various oral infections and aspiration pneumonia), with beta-lactam/beta-lactamase inhibitor combinations emerging as first line drugs next to metronidazole [1,93]. It is also important to realise that different susceptibility testing methods are appropriate for various needs and end points, e.g., whether it is performed in a diagnostic laboratory of a hospital, for epidemiological/surveillance purposes in a reference laboratory or in a research institution [94].

Some organisations govern the standards and practices for the antimicrobial susceptibility testing of bacteria: the Clinical Laboratory Standards Institute (CLSI; previously NCLLS) [95], the European Committee for Antimicrobial Susceptibility Testing (EUCAST; operating as a standing committee in the European Society of Clinical Microbiology and Infectious Diseases) [96], the British Society for Antimicrobial Chemotherapy (BSAC; which has since harmonized its criteria with EUCAST standards [97,98]) and the Deutsches Institut für Normung (DIN; German Institute for Standardization) [99]. The ESCMID Study Group for Anaerobic Infections (ESGAI) [100] and the Anaerobe Society of Americas (ASA) [101] play an equally important role in the facilitation of proper practice in the case of these bacteria.

Since there is no specified testing method for anaerobes by EUCAST, the standard procedures established by CLSI (currently M11-A8) are most frequently used [102]. According to this document, broth microdilution and agar dilution are the reference methods for this group of bacteria, although different methods are also accepted if their uniformity is confirmed with the reference method. CLSI also recommends that at least 100, randomly selected isolates of a given genera should be tested for their resistance patterns, to acquire adequate information on the regional (hospital) patterns of resistance [102]. Irrespective of the method used for the susceptibility testing on anaerobic bacteria, these all share the following: (i) without the proper (stable) level of anaerobiosis during the incubation period, the results cannot be interpreted [103]; (ii) since the generation time of these bacteria is longer than their aerobic counterparts, results of the susceptibility testing can only be interpreted after 48–72 h (or more); (iii) extensive quality control and staff experience is required [94]; (iv) breakpoints determined by CLSI and EUCAST do not always match, making interpretation of the susceptibility results difficult [102,103].

### 4.2. Disk Diffusion Method (Kirby-Bauer Method) 

Disk diffusion susceptibility testing is an easy-to-perform and cost-effective technique developed by EUCAST, their standardized methods and clinical breakpoints were embraced by clinical microbiology laboratories in Europe as well as in other parts of the globe [104]. Due to the popularity of this method, several studies (by EUCAST or otherwise) aiming at the evaluation of the suitability of this technique for the use in routine anaerobic bacteriology (mainly aiming at *B. fragilis* group isolates and “fast-growing” anaerobic strains) were performed [105,106]. Although some of these studies showed promising results in reproducibility (the inhibition zone diameters correlated well with the MICs in the case of metronidazole, imipenem, moxifloxacin and tigecycline), in other cases intermediate (I) and susceptible (S) isolates had overlaps in their inhibition zone diameters (in the case of beta-lactam/beta-lactamase inhibitor combinations and cefoxitin) [107]. Since the criteria for the use of this method has only been determined on aerobic bacteria so far, antimicrobial susceptibility testing of anaerobes using the disk diffusion method is not recommended in the routine lab yet and it is only used in a research setting or for preliminary screening for the antibacterial activity of different bioactive compounds [105]. However, “identification” disks containing specific antibiotics (usually colistin, kanamycin, metronidazole, penicillin and vancomycin are used) and sodium polyanethole sulfonate (SPS) are routinely used in the clinical microbiology laboratories for the presumptive identification of these bacteria [5].

### 4.3. Broth Microdilution Method

Broth microdilution assay is usually carried out using 96-well microtiter plates, where two-fold serial dilutions of the antibiotics are made. Commercial and in-house preparations are both available and the latter is more appropriate for the particular needs of the laboratory (various drugs can be tested in different concentrations), using only small volumes of reagents and media (Brucella broth supplemented with 5% lysed horse blood, 5 μg/mL hemin and 1 μg/mL Vitamin K1; according to CLSI recommendations) in the process. The results can be interpreted visually or with the help of a photometer. The results of the broth microdilution method are reported in Minimum Inhibitory Concentration (MIC) values, or the lowest concentration of antibiotics that stopped bacterial expansion. The commercially available standard broth microdilution panels are the breakpoint panels, in which only one or a few concentrations of each antimicrobial agent are tested in a single panel. Unfortunately, this method is not an all-round solution for anaerobic bacteriology, since apart from *Bacteroides* spp. the growth of other species is not consistent in broth (homogenous growth cannot be attained, which leads to reporting false susceptibility, spore-forming bacteria with no visible growth can also be falsely reported as susceptible). Another significant drawback of this method is that it can only be performed in an anaerobic glove box (for the appropriate level of anaerobiosis to be maintained), therefore its use in the clinical microbiology lab is limited [108]. 

### 4.4. Agar Dilution Method

Agar-dilution method is currently the *gold standard* for the antimicrobial susceptibility testing of anaerobic bacteria. During this procedure, nutrient agar plates (Brucella Agar supplemented with 5% laked sheep blood, 5 μg/mL hemin and 1 μg/mL Vitamin K1; according to CLSI recommendations) are made with various antibiotics incorporated in the media in different concentrations, following with the inoculation of the plates with a standardized number (inocula of 0.5 McFarland standard from a pure culture incubated for 48 h) of bacterial cells, usually with the help of a Steers-Foltz replicator device. After incubation of the plates for 48–72 h in anaerobic atmosphere, the plates are read and compared visually. Based on this method, the lowest concentration of antibiotic that inhibited the growth of the given bacterial strain is the MIC. Agar-dilution is an labour-intensive method recommended for anaerobic reference laboratories [23,102].

### 4.5. Gradient Tests (E-Test, Spiral Gradient Test)

Gradient tests such as the E-test are the most frequently used in hospital laboratories for performing anaerobic susceptibility testing. During the procedure, a plastic/paper strip (containing a pre-determined amount of antibiotic in a gradient fashion and a corresponding scale for MIC determination) is placed on the plate, after inoculation with the sample. It is recommended to dry the agar plates before applying the strips, to prevent excess moisture to change the characteristic concentration gradient. The zone of inhibition will present itself in an elliptical shape (hence the ‘E’ in the name of the test), the MIC should be read at the point where the zone of inhibition intersects the strip. The MIC reading of the E-test is independent of the number of colony forming units (CFU) of the inoculated sample, therefore the results should be considered reliable, and there is good correlation with the reference method. E-test can be used for the detection of heteroresistance (e.g., in the case of imipenem resistance of *B. fragilis* or in the case of metronidazole resistance of *C. difficile* and *B. fragilis*) as well [109,110]. It is easy to perform and it is especially useful for the testing of a small number of isolates or only one antibiotic in laboratories without a steady flow of numerous anaerobic isolates. 

The spiral gradient endpoint (SGE) technique is a special susceptibility testing method, which entails the use of a special agar plate, on which an apparatus deposits a specific amount of antibiotic stock solution in a spiral pattern [111]. By doing so, the concentration of the antibiotic is decreasing from the centre of the plate, creating a concentration gradient. After the inoculation of the plate with the relevant bacteria in a radial manner (thus, a single plate can be inoculated with multiple isolates and their susceptibilities to a single antibiotic can be determined simultaneously), the extent of growth is marked and the distance of the colonies from the centre of the plates is recorded. This data is then entered into a software (usually supplied by the manufacturer) which takes into account the physico-chemical characteristics of the antibiotic when determining the MIC related to the isolate in question. The emergence of resistant mutants can also be detected with this technique (colonies growing beyond the endpoint can be observed), while clumping of the bacteria was described (making interpretation difficult) when high-density inocula was used [112]. This method is becoming more and more popular because it’s easy-to-use and it showed favourable results, when compared with the results of agar dilution, however it is similarly high-priced to the reference method [111,113]. The lack of commercially available designs based on this method and the additional need to calculate the MIC from the observed results are some of the other drawbacks of this method [23].

### 4.6. Examples of Resistance Detection Using Phenotypic and Genotypic Methods

Some rapid tests are available for the detection of β-lactamase enzyme production of bacteria. Such methods, e.g., nitrocefin disks are practical due to the ease-of-use and the express results they provide. These colorimetric assays (a positive result is read if the disk changes to an orange or red colour in 5–60 min) are particularly useful if penicillin or ampicillin is the desired therapy [114]. During the interpretation of the results, it should be kept in mind that a negative result does not rule out resistance due to other mechanisms (e.g., changes in permeability due to poring loss or efflux pumps, modifications of PBPs) [115]. Performing this test is unnecessary on *B. fragilis* isolates, as the overwhelming majority produces β-lactamase enzymes [50]. Novel methods such as chromogenic media also present practical solutions for the use in the routine laboratory. An attempt for the development of such media was the *Bacteroides chromogenic agar* (BCA), in which 3,4-cyclohexenoesculetin-β-D-glucoside was included in the media instead of esculin to target the β-glucosidase activity of *B. fragilis*. In a precipitation reaction between the hydrolysed substrate with iron salts added to the media, an insoluble black precipitate appears, which allows for the differentiation of *B. fragilis* colonies from a polymicrobial isolate. Additionally, supplementation of BCA media with the appropriate concentrations of meropenem or metronidazole allows for the rapid detection of resistant strains of *B. fragilis* (e.g., from faeces), although this method is not yet routinely used in diagnostic laboratories [116].

Detection of resistance genes using polymerase chain reaction is a reliable method of identifying various antibiotic resistance determinants (for example: *nim* genes responsible for metronidazole resistance, *erm* genes conferring resistance to the macrolide-lincosamide-streptogramin (MLS) group of antimicrobials, the *cfiA* gene of carbapenem resistance, *gyr* and *parC* implicated in quinolone resistance, *cat* gene of chloramphenicol resistance, various *tet* genes making the microorganism resistant to tetracycline etc.) [23,117]. The end point would be the development of a multiplex PCR or a similar complex system, which would hopefully be able to identify multiple genes of resistance at once [117]. However, the development of similar methods is hindered by the fact that the presence of the resistance determinant genes in the genome does not automatically confer resistance to the antibiotics in question. This phenomenon has been well defined in the case of *B. fragilis* group isolates, harbouring “silent” *cfiA* or *cepA* genes (requiring specific insertion sequence (IS) elements to be activated), thus their genotypic resistance did not reflect their phenotypic resistance (i.e., their in vitro antimicrobial susceptibility patterns) [23,117,118].

Various experimental studies were conducted to test the potential future applications of matrix-assisted laser desorption/ionization time-of-flight (MALDI-TOF) mass spectrometry in the detection of various resistance determinants in anaerobes. In these studies, *B. fragilis* was predominantly used as a test organism for the detection of carbapenem resistance (as one of the most worrying resistance trends) from standard laboratory strains and later from clinical samples, such as blood cultures [118,119,120]. There is great interest in MALDI-TOF MS-based resistance determination, due to the superior speed of communicating the results, not to mention that these tests could be implemented on the mass spectrometry devices already purchased (with the addition of the appropriate database for detection). The detection of beta-lactamase production is also possible with the use of this technology. The hydrolysis of beta-lactam antibiotics by these enzymes results in a molecular mass shift, which can be detected by a MALDI-TOF MS-based assay, although due to its time-consuming nature, this method is not yet widely used [121,122,123]. 

Whole-genome sequencing for the prediction of antimicrobial resistance is a novel and emerging research area, the development of which is further facilitated by the emergence of reasonably-priced WGS systems [124,125,126,127]. Detection of known antibiotic resistant determinant genes from the bacterial strain isolated from the patients allows for a culture-independent method to predict the susceptibility pattern of microorganisms. This makes WGS a useful tool for surveillance purposes, even in the case of drugs that are usually not included in classical (phenotypic) susceptibility testing routines (e.g., in drug development). [74,128,129]. Additionally, resistant phenotypes caused by dissimilar genetic backgrounds can be differentiated [124,125]. According to recent studies on selected species groups, the concordance rate between the presence of antibiotic resistance genes and the actual antibiogram (phenotype) of the microorganism is between 72–99%, although the use of this method is still in its experimental stages, due to the lack of properly developed reference libraries of bacterial resistance determinants (seeing that only the resistance genes included in the web-based databases will be recognized-a common disadvantage of molecular diagnostic methods) [129,130].

## 5. Antibiotic Resistance in Anaerobic Bacteria: The Importance of Surveillance

Nowadays the treatment of infections caused by anaerobic bacteria (or a mixed infection having an anaerobic component) rests upon the following antimicrobials: penicillins (ampicillin/ticarcillin), beta-lactam-beta-lactamase inhibitor combinations (amoxicillin/clavulanic acid, ticarcillin/clavulanic acid, ampicillin/sulbactam, piperacillin/tazobactam), cefoxitin and cefotetan (cephamycins), clindamycin, tigecycline, moxifloxacin, macrolides (azithromycin, clarithromycin), chloramphenicol and metronidazole [1,23,93,131]. 

Some data available on the resistance trends of anaerobes (apart from hospital-level or regional surveillance studies conducted where resources were available) is restricted to the studies published by anaerobic reference laboratories or major trans-national collaborations [23,132,133,134,135,136]. Although the variations between different geographical regions are notable, common tendencies can be observed.

While three decades ago the antibiotic susceptibility pattern of anaerobic bacteria was straightforward, nowadays we cannot so easily predict the efficiency of the chosen empirical therapy [91]. Clinicians can no longer “expect” certain drugs to work in anaerobic infections because they showed potent activity before [137]. Though not at the same speed as some other microorganisms (e.g., carbapenem-resistant *Acinetobacter baumannii*, *Pseudomonas aeruginosa* or members of the *Enterobacteriaceae* [138]) resistance has steadily increased among anaerobes during the last 30 years. 

With the use of broad-spectrum antimicrobials in addition to the suitable surgical measures, the issue of emerging resistance of anaerobic bacteria was maybe less obvious [93,131]. But if we observe the data from the (inter)national surveillance reports from Europe (Table 6) [139,140,141,142,143,144,145,146,147] and the United States (Table 7) [95,135,144,145,146,147], the same trends can be discovered: steadily growing resistance to all classes of antimicrobial agents, with some of them rendered completely useless. Several publications report the emergence of multidrug resistant anaerobic Gram-negative bacteria (especially within the *B. fragilis* group isolates), harbouring multiple resistance genes or with a combination of intrinsic and acquired resistance mechanisms [80,85,147,148,149,150,151]. In these cases, the bacteria were usually termed multidrug resistant if they were resistant to 3–4 antibiotic classes besides metronidazole (due to *nim* nitroimidazole resistance genes) and the carbapenems (a metallo-β-lactamase encoded by *cfiA*/*ccrA* genes) [79]. Mobile genetic elements (plasmids containing resistance determinants, insertion sequence elements, transposons) have a significant role in the spread of the multidrug resistant phenotype in anaerobes. 

The significance of the abovementioned tendencies is further underlined by the fact, that treatment failure has been described in empirical treatment in cases of anaerobic bacteraemia, as result of a multidrug resistant *B. fragilis* infection [9,84,152,153,154,155]. What makes this problem even more insidious is the fact that the correlation between the presence of multidrug resistant anaerobic bacterial strains and clinical failure is hard to prove. Mixed infections that include anaerobic bacteria often improve by drainage or surgical debridement [2]. The well-being of the patient (chronic diseases, immunosuppression) and the effect of the administered antibiotics on the aerobic component of the infection could further influence clinical outcome. Lastly, if the specimen collection and processing of the anaerobic isolate was not appropriate, the suspected microorganism cannot be cultivated, even if there is strong clinical suspicion of its presence [63].

A summary of some of the important resistance mechanisms (and their genetic determinants) is presented in Table 8. While the growing problem of resistance stems from the fact that resistance is gradually developing against drugs that once were efficacious in treatment (analogous to the trends observed in other bacteria, e.g., *Enterobacteriaceae*) it is important to be aware that anaerobes–owing to their biological nature–are inherently resistant to some antimicrobials. For example, all anaerobic bacteria are resistant to the aminoglycosides, since the antibiotic cannot reach its target molecule (30S subunit of the ribosome). The uptake of an aminoglycoside drug by a bacterial cell is a two-step process, requiring the presence of oxygen- or nitrogen-dependent electron transport chains, a mechanism that all anaerobes lack, therefore making the impact of this antibiotic class limited. Anaerobes exhibit similar innate resistance towards fosfomycin, trimethoprim, aztreonam and all 1st and 2nd generation quinolones through a variety of processes, therefore using these drugs in therapy would be ill-advised [156,157,158]. Other instances of intrinsic resistance are species dependent, like in the case of metronidazole resistance of various Gram-positive anaerobes (*Actinomyces* spp., *Lactobacillus* spp., Bifidobacterium spp., *Propionibacterium* spp.), the macrolide and rifampin resistance in *Fusobacterium nucleatum* and *F. mortiferum* and the resistance against cephalosporins in *C. difficile*. 

*Clindamycin* was considered the gold standard for the treatment of anaerobic infections some 40–50 years ago but with the emergence of high levels of resistance among *C. difficile* (~70%), *B. fragilis* group (30–40%), *Prevotella* spp. (10–40%), other related anaerobic Gram-negative bacteria (~10%) and *Peptostreptococcus* spp (~10%), this drug lost its significance as a first-line drug. Clindamycin resistance of chromosomal origin is linked to tetracycline resistance determinants, although their presence in plasmids and conjugative transposons was also described. Several studies showed that *macrolides* (azithromycin, clarithromycin) are potent agents against some anaerobic bacteria, although their bacteriostatic effects hinder their usefulness in serious infections. Resistance to the macrolide-lincosamide-streptogramine-group of antimicrobials manifest uniformly (which can be attributed to various *erm* genes): due to the methylation of two specific adenine residues of the 23S rRNA, the effective binding of the drugs to ribosome is prevented [159,160,161,162,163]. 

*Chloramphenicol* was a drug-of-choice for the treatment of serious anaerobic infections (especially if the central nervous system was involved), nowadays is an infrequently used drug, due to its considerable toxicity and serious reversible (haemolytic anaemia, optic neuritis, bone marrow suppression) and irreversible (fatal aplastic anaemia) side effects. Resistance against chloramphenicol is mostly plasmid-mediated, by inactivation of the active drug through acetylation or nitro-reduction [164,165]. 

*Quinolones* were generally considered ineffective against anaerobic bacteria due to their bacteriostatic effects and the poor drug penetration to the target sites, together with the low affinity for the target enzymes, owing to point mutations in the *gyrA-B* (Topoisomerase II) and *parC* genes (Topoisomerase IV). Additionally, quionolone resistance determining regions (QRDR; like that found in *E. coli*) were observed in several anaerobic species, with an increased expression of efflux proteins. These quinolone resistance mechanisms were mostly observed in *B. fragilis* group, *C. perfringens* and *C. difficile* isolates [166,167,168,169]. Moxifloxacin is a new drug in this group of antimicrobials, with FDA approval for complicated skin and skin structure infections, with promising anti-anaerobe activity.

Resistance against *β-lactam antibiotics* is very common among anaerobes (predominantly due to the production of β-lactamase enzymes), although there are significant variations to the levels of resistance between these bacteria. Almost 100% of *B. fragilis* isolates are resistant to penicillin G, since they constitutively produce a chromosomally-encoded penicillinase enzyme; this is around 50% in the case of *Prevotella* spp. and between 8–17% for *Porphyromonas* spp. While *Cl. perfringens* isolates are ~100% susceptible to penicillin G (ampicillin), resistance has emerged in non-perfringens clostridia (e.g., *Cl. clostidiforme*, *Cl. butyricum, Cl. ramosum*) [170]. The production of a class 2e cephalosporinase (encoded by the *cepA* and *cfxA* genes; *B. fragilis*) confers resistance against cefoxitin and cefotetan; this enzyme is inhibited by β-lactamase enzyme inhibitors (clavulanic acid, sulbactam, tazobactam). Cephamycins such as cefoxitin (with a 7α-methoxyl side chain) and cefotetan (with an N-methylthiotetrazole side chain) can still be considered appropriate therapy for anaerobic infections (in contrast to cephalosporins) but consulting regional epidemiological data about resistance levels is recommended. An emerging issue is the appearance of a Zn^2+^-metallo-β-lactamase enzyme (essentially a carbapenemase, encoded by the *cfiA*/*ccrA* genes; it is inhibited by EDTA) in *B. fragilis*. These isolates are resistant against the carbapenem antibiotics, which are usually preserved as last-resort drugs in serious, life threatening infections. It has been described that more *Bacteroides* isolates harbour these resistance genes than the number of isolates with a phenotypic resistance. The probable cause of this phenomenon is that these genes are not expressed in levels (“silent genes”), where they could exhibit decreased susceptibility carbapenems. However, with a one-step mutation, or with the insertion of an IS element, the strain can easily mutate to become resistant. Reduced permeability of the drugs through the outer membrane in some *Fusobacterium* spp., *Porphyromonas* spp. and *Bacteroides* spp. can also lead to decreased susceptibility. PBP alterations (leading to reduced binding affinity of these drugs) were described in several Gram-positive anaerobic cocci. It can be said that, for now, β-lactam antibiotics (especially beta-lactam/beta-lactamase inhibitor combinations and carbapenems) still represent the frontline of treatment in mixed aerobic-anaerobic infections [23,93]. Penicillin G is still a useful drug in our disposal and its use is appropriate if the strain in question is susceptible. Beta-lactam-beta-lactamase inhibitor combinations (with resistance levels of 0.5–10%) and carbapenems (0–2%) should be considered safe choices for empiric therapy [151,171,172,173,174,175,176,177,178,179,180,181]. The importance of surveillance is extremely important related to this group of drugs, with the number of resistant strains steadily growing and with the emergence of multidrug resistant anaerobes (note: some carbapenem and metronidazole resistance elements share mobile genetic elements) [79,149]. Such a pathogen of increasing importance is *Sutterella wadsworthiensis*, with increasing resistance rates to piperacillin/tazobactam, cefoxitin, clindamycin and metronidazole [182,183]. 

*Metronidazole* resistance is common in various Gram-positive anaerobic rods (*Actinomyces* spp., *Propionibacterium* spp., *Lactobacillus* spp.), while the prevalence of resistant Gram-positive cocci and Gram-negatives is usually very low (<1%). Metronidazole (and other related drugs) are a part of the 5-nitroimidazole group drugs and are one of the most important agents for the treatment of anaerobic infections. Metronidazole is a pro-drug: for the desired antimicrobial activity, it must be reduced for the release of toxic nitroso-residues (PFOR; pyruvate: ferredoxin oxidoreductase and the nitroreductase enzymes) that are extremely reactive (able to damage DNA, proteins and membranes of bacteria). Some consider metronidazole an example for the “ideal antibiotic model” [184]. Resistance to this drug is conferred by the *nim* genes (*nimA-J*) coding for a nitroimidazole-reductase: this transforms the pro-drug to a non-toxic amino-imidazole, with no antimicrobial activity. These *nim* genes share around 70% homology (*B. fragilis* group), apart from *nimI* (*Prevotella* spp.). *nimB* has been associated with moderate to high level metronidazole resistance. Other mechanisms of reduced susceptibility include lower levels of the PFOR enzyme (a significant decrease of the reducing power of the microorganisms) and the induction of lactate dehydrogenase (LDH) activity. These resistance determinants are predominantly found in the chromosome but their presence has been described in transferable plasmids and IS elements as well, with some experts fearing the rapid dissemination of these genes [149,150,185,186,187,188,189,190,191,192,193,194]. 

*Tetracycline* resistance is very common in anaerobic isolates (>95% of *Bacteroides* spp., >50% of *Prevotella* spp., *Fusobacterium* spp. and *Clostridium* spp.) to the point that the use of these drugs is not advised. Among the resistance mechanisms, drug efflux (*tetA-E*, *tetK-L*), ribosomal protection (*tetM*, *tetQ*, *tetW*) and oxidation (*tetX*) can be found. The *tet* genes are induced by sub-inhibitory tetracycine exposure, stimulating the spread of resistance trough conjugative transposons [160,170,195,196,197]. Tigecycline (the tert-butylglycamido-derivative of minocycline) is a novel drug in this antibiotic class, having FDA approval for soft tissue and intra-abdominal infections. 

Efflux pumps are important determinants of antimicrobial resistance both in aerobic and anaerobic bacteria, their over-expression can often be associated with therapeutic failure. These transmembrane proteins can bind and expel various substrates and xenobiotics (such as antibiotics) before they could reach their target site or protein. Several efflux pumps have been characterized among anaerobic bacteria: the BexA of *B. thetaiotaomicron* (a transporter of the multi-antimicrobial extrusion protein [MATE] superfamily) the bmeABC1–16 (members of the resistance-nodulation-cell division [RND] superfamily, analogous to the AcrAB-TolC tripartite efflux systems of *E. coli*) and TetA-E efflux transporters (members of the major facilitator superfamily [MFS] superfamily) of *B. fragilis*, bcrABD (an ATP-binding cassette [ABC] transporter) of *Cl. perfringens* and XepCAB (member of the RND superfamily) of *P. gingivalis* [198,199,200,201]. Experimental studies have shown that knocking out one or more of these efflux proteins has a remarkable effect on the MICs of several antibiotics, although due to the redundancy of these proteins in the bacterial genome, other transporter mechanisms usually compensate for the loss of the selected transporter. An emerging therapeutic strategy is the use of efflux pump inhibitors (EPI) as adjuvant compounds, although these compounds were only tested extensively in laboratory conditions so far [198,202]. 

(Since this is not the main topic of our review, the information on the various modes of antibiotic resistance among anaerobes presented above is a brief summary of the available literature. For further reading on this topic, see [11,23,93,200,203]).

## 6. Conclusions

Anaerobic microorganisms are now widely accepted as significant pathogens in human diseases, as such, the proper diagnosis and treatment of these infections are important healthcare priorities. The emergence of antimicrobial resistance in anaerobic bacteria is a discernible phenomenon, which deserved the attention of people working in diagnostic microbiology and infectious disease treatment. Microbiology laboratories should perform antimicrobial susceptibility testing to the best of their abilities, taking into account the number of incoming anaerobic isolates, as well as monetary considerations of the given institution. This incentive is important to broaden the information on regional, national and global patterns of resistance of anaerobes. Based on the plethora of literature available, the slow but steady increase of metronidazole and carbapenem resistance isolates is a cause for concern, especially if we observe what happened to the efficiency of other agents (like clindamycin) in the previous 20–30 years.

## Figures and Tables

**Table 1:** Summary of the most important genera of anaerobic bacteria [3,6].

Gram-Positives		Gram-Negatives	
Cocci	Rods	Cocci	Rods
*Anaerococcus*	*Actinomyces*	*Acidaminococcus*	*Bacteroides*
*Atopobium*	*Bifidobacterium*	*Megasphera*	*Bilophila*
*Coprococcus*	*Clostridium*	*Veillonella*	*Butyrivibrio*
*Finegoldia*	*Eubacterium*		*Centipeda*
*Gaffkya*	*Lactobacillus*		*Desulfonomonas*
*Gallicola*	*Propionibacterium*		*Fusobacterium*
*Parvimonas*			*Leptotrichia*
*Murdochiella*			*Mitsuokella*
*Peptococcus*			*Mobiluncus*
*Peptostreptococcus*			*Porphyromonas*
*Peptoniphilus*			*Prevotella*
*Ruminococcus*			*Selenomonas*
*Sarcina*			*Succinimonas*
			*Succinivibrio*
			*Sutterella*
			*Wolinella*

**Table 2:** Species of the *Bacteroides fragilis* group [50].

Bacteroides				Parabacteroides
*B. acidifaciens*	*B. eggerthii*	*B. massiliensis*	*B. sartorii*	*P. chartae*
*B. barnesiae*	*B. faecis*	*B. nordiia*	*B. stercoris*	*P. distasonis*
*B. caccae*	*B. finegoldii*	*B. oleiciplenus*	*B. thetaiotaomicron*	*P. goldsteinii*
*B. cellulosilyticus*	*B. fluxus*	*B. ovatus*	*B. uniformis*	*P. gordonii*
*B. chinchillae*	*B. galacturonicus*	*B. plebeius*	*B. vulgatus*	*P. johnsonii*
*B. clarus*	*B. gallinarium*	*B. propionifaciens*	*B. xylanisolvens*	*P. merdae*
*B. coagulans*	*B. graminisolvens*	*B. pyogenes*	*B.xylanolyticus*	
*B. coprocola*	*B. helcogenes*	*B. rodentium*	*B. zoogleoformans*	
*B. coprophilus*	*B. heparinolyticus*	*B. salanitronis*		
*B. dorei*	*B. intestinalis*	*B. salyersiae*		

**Table 3:** Illnesses caused by human pathogenic species of the *Clostridium* genus [6,7,17,18,87,88,89,90].

Pathogen	Disease
*C. argentinense* *C. baratii* ***C. botulinum*** *C. butyricum*	*Botulism*
*C. bifermentans* *C. fallax* *C. histolyticum* *C. novyi* ***C. perfringens*** *C. sordelii*	*Soft tissue infections (gas gangrene, suppurative myonecrosis, cellulitis)* *Food poisoning* *Enteritis necrotisans* *Endometritis* *Sepsis*
***C. tetani***	*Tetanus*
***C. difficile***	*Antibiotic-associated diarrhoea* *Pseudomembranous colitis* *Toxic megacolon*
*C. clostridioforme* *C. innocuum* *C. sporogenes* *C. tertium*	*Opportunistic infections*

The bacteria in bold represent the most prevalent species in the group.

**Table 4:** List of anaerobic bacteria where routine susceptibility testing is recommended [23].

*Bacteroides fragilis* group
*Bilophila wadsworthia*
*Clostridium innocuum*
*Clostridium perfringens*
*Clostridium ramosum*
*Fusobacterium* spp.
*Prevotella* spp.
*Sutterella wadsworthensis*
novel clinically important anaerobes (where surveillance data is not available)

**Table 5:** Antimicrobial agents that should be included in the routine susceptibility testing on anaerobic isolates [11,23,92].

Obligatory ^a^	Accessory ^b^
5-nitroimidazole group drugs ^c^	cefoxitin
penicillins	moxifloxacin
beta-lactam-beta-lactamase inhibitore combination ^d^	tigecycline
clindamycin	Vancomycin ^f^
Carbapenems ^e^	Fidaxomycin ^f^

^a^ Accurate if the bacteria have no intrinsic resistance to the agent; ^b^ If the agent has the appropriate indications by the FDA/EMA for the infection in question; ^c^ Including metronidazole, tinidazole, secnidazole, onidazole; ^d^ Including amoxicillin/clavulanic acid, ticarcillin/clavulanic acid, ampicillin/sulbactam, piperacillin/tazobactam; ^e^ Including imipenem, meropenem, ertapenem and doripenem; ^f^ Relevant in the case of *Clostridium difficile*.

**Table 6:** Resistance trends of clinical *Bacteroides* isolates in Europe between 1990–2010 (expressed as the percentage of resistant isolates) [94,135,145].

Europe	AMP	AMX/CLA	PIP/TAZ	FX	IMP	CLI	TET	MET	CIP	MXF
**Breakpoints (mg/L)**	**32/64**	**8**	**128**	**32/64**	**4/16**	**4/8**	**4**	**8/32**	**4**	**8**
1990	16.0%	1.0%	-	3.0%	0.3%	9.0%	64.0%	0.0%	56.0%	-
2000	99.3%	-	1.0%	6.0%	0.7%	15.0%	-	0.5%	-	9.0%
2010	98.2%	10.4%	10.3%	17.2%	1.2%	32.4%	-	0.5%	-	13.6%

AMP: ampicillin; AMX/CLA: amoxicillin/clavulanic acid; PIP/TAZ: piperacillin/tazobactam; FX: cefoxitin; IMP: imipenem; CLI: clindamycin; TET: tetracycline; MET: metronidazole; CIP: ciprofloxacin; MXF: moxifloxacin.

**Table 7:** Resistance trends of clinical *Bacteroides* isolates in the United States between 1990–2009 (expressed as the percentage of resistant isolates) [139,140,141,142].

USA	AMP	AMP/SUL	PIP/TAZ	FX	IMP	CLI	TET	MET	CIP	MXF
**Breakpoints (mg/L)**	**-**	**32**	**128**	**16/64**	**8/16**	**4/8**	**-**	**16**	**-**	**8**
1990	-	-	-	11.0%	0.0%	5.0%	-	0.0%	-	-
1997/2004	-	2.6%	0.5%	10.3%	0.4%	25.6%	-	0.0%	-	34.5%
2009 ^a^	-	6.5%	0.8%	10.9%	0.6%	35.2%	-	-	-	50.1%

AMP: ampicillin; AMP/SUL: ampicillin/sulbactam; PIP/TAZ: piperacillin/tazobactam; FX: cefoxitin; IMP: imipenem; CLI: clindamycin; TET: tetracycline; MET: metronidazole; CIP: ciprofloxacin; MXF: moxifloxacin. ^a^ calculated from the average of the resistance percentages of the individual *Bacteroides* strains investigated.

**Table 8:** Examples for antimicrobial resistance mechanisms exhibited by anaerobes [11,23,93,200,203].

Antibiotic Class	Mechanism of Resistance	Genes or Enzymes Implicated	Examples of Microorganisms
Aminoglycosides	Lack of O- or N-based electron transport systems; Unable to reach target ribosome subunit (30S)		All anaerobes
β-lactams	β-lactamase enzymes:		
	Penicillinases		*Clostridium* spp. *Fusobacterium* spp., *Prevotella* spp., *Porphyromonas* spp.
	Cephalosporinases	*cepA*, *cfxA*	*B. fragilis* gp
	Metallo-β-lactamases	*cfiA*, *ccrA*	*B. fragilis* gp.
	Reduced affinity to target molecule	PBP1–2 alterations	Anaerobic Gram-positive cocci, *B. fragilis* gp.
		PBP3 (aztrenonam)	All anaerobes
	Loss of porin channels		*B. fragilis* gp.
Chloramphenicol	Inactivation		
	Acetylation	cat	*B. fragilis* gp.
	Nitro-reduction		*B. fragilis* gp.
Clindamycin	Methylation of the 23S rRNA	*ermF*, *ermG*, *ermS*	*B. fragilis* gp.
		*ermB, ermF, ermG, ermFG,*	*Prevotella* spp.
		*ermF*	*Porphyromonas* spp.
		*ermB*, *ermQ*	*Cl. difficile*
		*ermP*, *ermQ*	*Cl. perfringens*
	Inactivation		*B. fragilis* gp.
Macrolides	Methylation of the 23S rRNA	*ermA*, *ermB*, *ermF*, *ermG*, *ermQ*, *ermTM*	*F. magna*, *P. tetradius*, *P. anaerobius*
			
Metronidazole	Intrinsic		Gram-positive anaerobic bacteria
	Reduction of the drug by nitroimidazole reductase	*nimA-H*	*B. fragilis* gp., *Veillonella* spp.
		*nimI*	*Prevotella* spp.
	Reduced uptake of the drug		*B. fragilis* gp.
	Increase in LDH activity		*B. fragilis* gp.
Quinolones	Mutations in target enzymes		
	DNA-gyrase (Topoisomerase II)	*gyrA*, *gyrB*	*B. fragilis*, *Cl. perfringens*, *Cl. difficile*
	Topoisomerase IV	*parC*	*Cl. difficile*
Tetracyclines	Ribosomal protection	*tet(Q)*	*B. fragilis* gp.
		*tet(M)*, *tet(W)*	*Fusobacterium* spp.
		*tet(M)*, *tet(Q)*, *tet(W)*	*Prevotella* spp.
	Ribosomal modification	*tetA(P)*, *tetB(P)*	*Clostridium* spp.
	Efflux pumps	*tetA-E*	*B. fragilis* gp.
		*tetK-L*	*Peptostreptococcus* spp., *Veillonella* spp.
	Enzymatic degradation (oxidative)	*tetX*	*B. fragilis* gp.

## Abbreviations

AAD	antibiotic associated diarrhoea
ABC	ATP-binding cassette
AGNB	anaerobic Gram-negative bacilli
AIDS	acquired immunodeficiency syndrome
AMX/CLA	amoxicillin-clavulanic acid
AMP/SUL	ampicillin-sulbactam
ASA	Anaerobe Society of the Americas
AST	antimicrobial susceptibility testing
ATP	adenosine-triphosphate
BBA	Brucella blood agar
BBE	Bacteroides bile esculin agar
BfPAI	*Bacteroides fragilis* pathogenicity island
BL-BLIC	beta-lactam-beta-lactamase inhibitor combination
BSAC	British Society for Antimicrobial Chemotherapy
BV	bacterial vaginosis
Caco-2	Human epithelial colorectal adenocarcinoma
CCFA	Cefoxitin-cycloserine fructose agar
CNA	Colistin-nalidixic acid agar
CIDT	culture-independent diagnostic testing
CIP	ciprofloxacin
CLI	clindamycin
CLSI	Clinical and Laboratory Standards Institute
DIN	Deutsches Institut für Normung
DNA	deoxyribonucleic acid
EBV	Epstein-Barr virus
EDTA	ethylenediaminetetraacetic acid
EPI	efflux pump inhibitor
EMA	European Medicines Agency
ENRIA	European Network for Rapid Identification of Anaerobes
ESGAI	ESCMID Study Group for Anaerobic Infections
ETBF	enterotoxigenic *Bacteroides fragilis*
EUCAST	European Committe for Antimicrobial Susceptibility Testing
EYA	Egg-yolk agar
FDA	Food and Drug Administration of the United States
FX	cefoxitin
GLC	gas-liquid chromatography
IBS	irritable bowel syndrome
HCT-8	human ileocecal carcinoma
HGT	horizontal gene transfer
HT-29	human colon adenocarcinoma cell line
ID	identification
IS	insertion sequence
IMP	imipenem
KVLB	Kanamycin-vancomycin laked blood agar
LDH	lactate-dehydrogenase
MALDI-TOF MS	matrix-assisted laser desorption/ionization time-of-flight mass spectrometry
MATE	Multi-antimicrobial extrusion protein
MDCK	Madin Darby Canine Kidney cell line
MDR	multidrug resistant
MET	metronidazole
MIC	minimal inhibitory concentration
MLS	Macrolide-lincosamide-streptogramin B
MXF	moxifloxacin
NCLLS	National Committee for Clinical Laboratory Standards
PBP	penicillin-binding protein
PCR	polymerase chain reaction
PEA	Phenyletyl alcohol agar
PFOR	pyruvate:ferredoxin oxidoreductase
PID	pelvic inflammatory disease
PIP/TAZ	piperacillin-tazobactam
PRAS	pre-reduced anaerobically sterilised
QRDR	quionole resistance determining regions
RND	Resistance nodulation and division
rRNA	ribosomal ribonucleic acid
SCFA	short-chained fatty acid
SGE	spiral gradient endpoint
SOD	superoxide-dismutase
SCS	Schaedler blood agar
SPS	sodium polyanethol sulfonate
TET	tetracycline
WGS	whole-genome sequencing
